# Preparing House Officers for Emergencies in a Psychiatric Setting with Simulation-Based Learning

**DOI:** 10.1007/s40596-025-02217-4

**Published:** 2025-09-06

**Authors:** Matthew Tennant, Maggie Meeks, Ben Beaglehole, Chris Frampton, Jane Foley, Emily Ide, Dylan Hill, Carol Dean

**Affiliations:** 1 Te Whatu Ora, Christchurch, Canterbury New Zealand; 2 University of Otago, Christchurch, Canterbury New Zealand

**Keywords:** Simulation-based learning, Post-injection syndrome, Non-suicidal self-injury, Non-fatal hanging

## Abstract

**Objective:**

Simulation-based learning replicates real clinical events to provide an interactive learning experience. This allows training doctors to develop skills to manage complex scenarios or emergencies in a safe and containing environment. Many house officers report a lack of confidence in managing emergencies on a psychiatric ward. This study evaluates whether simulation-based learning provided at the start of the psychiatric placement increases the confidence of house officers managing emergencies in a psychiatric setting.

**Method:**

Simulation-based learning was developed and implemented for house officers beginning their psychiatric rotation. Three scenarios were developed with psychiatric trainees. These were managing a non-fatal hanging, non-suicidal self-injury, and olanzapine pamoate post-injection syndrome. Training was evaluated with a mixed methods approach.

**Results:**

Twenty-three house officers participated in simulation-based learning. After completing the training, participants were significantly more confident managing olanzapine pamoate post-injection syndrome (*p* < 0.001), non-suicidal self-injury (*p* < 0.001), and a non-fatal hanging (*p* < 0.001). Participants reported that simulations were effective because the scenarios were realistic, and the simulation was an immersive experience. They valued the opportunity to practice with the equipment and the focus on both physical and psychological components in the scenarios. The experience was more impactful because simulations were done alongside peers in a safe and contained training environment.

**Conclusion:**

Simulation-based learning was acceptable and increased the confidence of house officers managing emergencies in a psychiatric setting. Simulation-based learning can potentially improve the quality of care provided in psychiatric hospitals and the preparedness of new doctors entering these work environments.

The value of clinical experiences in developing skills as a doctor has never been in doubt. Simulation-based learning replicates real clinical events to provide an interactive learning experience. This allows training doctors to develop skills to manage complex scenarios or emergencies in a safe and containing environment while protecting service users from unnecessary risks [1]. It also allows the development of clinical skills, such as interdisciplinary collaboration and communication [2, 3].

A recent systematic review and meta-analysis concluded that simulations were an effective tool for psychiatric education [4]. But until recently, simulation-based learning had a limited role in psychiatric training, being used most often to teach communication skills and to test knowledge in the form of an objective structured clinical examination (OSCE) [5, 6]. There is growing evidence for the utility of simulation training for core psychiatric skills. For example, history taking, risk assessment, prescribing antipsychotics, Mental Health Act processes, and verbal de-escalation of agitated patients [7–12]. However, simulation-based learning aimed to build confidence in medical emergencies within a psychiatric setting is less common [13–15]. To our knowledge, simulation of a non-fatal hanging attempt in a psychiatric setting has not yet been evaluated. Yet, when starting on psychiatric placements, many house officers report a lack of confidence in managing emergencies on a psychiatric ward [15, 16].

This study evaluates whether simulation-based learning, which focuses on both physical and psychological components of an emergency, can increase the confidence of house officers managing emergencies in a psychiatric setting.

## Method

Social learning theory and experiential learning theory have been drawn upon in the development of a program using simulation-based learning to teach new doctors how to manage emergencies in a psychiatric setting. A critical realist approach underpins our evaluation, which takes both quantitative and qualitative perspectives. Critical realism in this paper means that we believe we can make inferences about the “real world” based on our observations, and that data from multiple research modalities are more meaningful (hence supporting a mixed methods approach). It supports a generative understanding of causation. That is to say, the context in which an intervention is applied is very important in understanding the intervention’s effectiveness, and so we have attempted to provide as much contextual detail in this paper as possible. Unlike some other qualitative approaches to analysis, critical realism allows for abductive reasoning [17]. Ethical approval was granted for this study by the University of Otago Human Ethics Committee (23/075). Participants provided written informed consent before entering the study.

### Development of Scenarios

We asked a group of seven psychiatric trainees what clinical scenarios they felt under-prepared to manage when they started their work in psychiatry. Based on their discussion, three scenarios were selected: managing a hanging attempt, deliberate self-harm, and post-injection syndrome. Each scenario had been experienced as clinicians by the trainees when they were working as house officers in a psychiatric hospital. These were uncommon scenarios that are difficult to teach in real clinical environments and were therefore suited to simulation-based learning. Simulations were then developed by a psychiatric trainee in the group who had experienced them in collaboration with experts in simulation-based learning, senior psychiatric nurses, and consultant psychiatrists. Full simulation protocols are available on request.

In scenario I, house officers are required to manage olanzapine post-injection syndrome while remaining aware of other potential causes of collapse in a psychiatric inpatient setting. In scenario II, house officers are required to assess a laceration of the forearm (non-suicidal self-injury) while demonstrating trauma-informed care principles with a service user diagnosed with complex PTSD in a psychiatric inpatient setting. In scenario III, house officers are required to utilize the appropriate cut down technique and perform an initial assessment of the airway and neurological function following a suicide attempt by hanging at a psychiatric hospital.

### Implementation of Simulation Training

In New Zealand, after completing their 6-year medical degree, doctors are employed as house officers within the public health system for at least 2 years before entering specialist training. During this period, they gain experience in various medical and surgical specialties on 3-month rotations. At our local hospital, most house officers do at least one rotation in psychiatry. Therefore, many of the house officers who work within the psychiatric hospital will eventually specialize in other areas of medicine.

Within the first fortnight of their 3-month psychiatry rotation, house officers were required to participate in simulation training. House officers were given background reading about all three scenarios so that they had the opportunity to broaden their knowledge before the simulation training. They were also informed that one of the scenarios involved a simulated hanging. If they believed that this could be traumatizing or re-traumatizing for them because of prior experiences, they were given the opportunity to discuss participation with a psychiatrist confidentially before the training and the option of withdrawing from the training. House officers were invited to participate in the research before attending the training, and they had the option of completing the training while not participating in the study, which evaluated the training.

Simulation training was run over a 2-hour period in small groups of three to six house officers plus three to five trainers. Two house officers volunteered to actively participate in each simulation. All the house officers were involved in the briefing and debriefing discussions. The debrief was held in a different room from the simulations so that the next scenario could be set up while participants were debriefing.

Before entering the simulation, a briefing orientated house officers to the scenario and the boundaries of the simulation. Psychological safety and clinician’s vulnerability were discussed, recognizing how traumatic experiences in the workplace can impact on their own mental health and professional development. Key learning points for the scenario were reviewed and questions arising from the pre-reading were answered so house officers were set up for success in the scenario.

Psychiatry registrars (senior resident doctors specializing in psychiatry) and psychiatric nurses were actors in the scenarios. Costumes, prosthetics, makeup, and props were used to make the simulations as immersive as possible. House officers who were not actively participating in the simulation observed their colleagues silently.

Debriefing followed the Promoting Excellence and Reflective Learning in Simulation (PEARLS) model [18]. This model utilizes learner self-evaluation and facilitates focused discussion and direct feedback to learners. The debriefing was led by a simulation educator, a consultant psychiatrist, a psychiatric registrar, and a senior nurse. The debriefing emphasized non-technical skills such as situational awareness and trauma-informed care. It also explored psychological aspects of the simulation, including countertransference.

### Data Collection and Analysis

Demographic information of participants was collected, including gender, ethnicity, medical experience (post-graduate year), and previous experience on a psychiatric ward. Participants were able to select multiple ethnicities. The WHO-5 self-reported wellbeing measure and the Rosenburg Self-esteem Scale were collected before the simulation training. The WHO-5 is scored from 0 to 25, where 0 is the worst possible wellbeing and 25 is the greatest wellbeing. When used as a screening tool for psychiatric disorders, a score below 13 indicates that further assessment is needed. The Rosenburg Self-esteem Scale is a self-reported scale of self-esteem. On this scale, 10–25 indicates low self-esteem, 26–29 indicates medium self-esteem, and 30–40 indicates high self-esteem. Confidence levels related to each emergency scenario were self-reported on a Likert scale questionnaire before and directly after the simulation training. Participants rated their confidence from 1 (less confident) to 5 (more confident). Because these scales recorded ordinal data, central tendency was reported with a median, and variability in the Rosenburg Self-esteem Scale and WHO-5 was reported using interquartile range. A Wilcoxon signed-rank test was used to evaluate whether changes in confidence were statistically significant. The primary outcome measure was self-reported confidence to manage an emergency in a psychiatric setting.

In addition, participants were asked to provide written feedback to the following questions on completion of the training: What factors from simulation training help to improve doctors’ confidence when confronted with an emergency on a psychiatric ward? What aspects of the simulation training were helpful? What aspects of simulation training could be improved? Any other comments about the training?

Responses were collected and copied into Microsoft Excel. The responses were systematically coded and then categorized and summarized based on the content analysis method described by Kleinheksel et al. [19]. This involved the author (MT) becoming immersed in the data to become familiar with the scope through several readings of the text. Then, codes (units of meaning) were assigned to the text based on the manifest content. Similar codes were then grouped together into categories and described.

## Results

Between January 2024 and October 2024, 36 house officers worked within the psychiatric service. Twenty-three participated in simulation training. All the house officers who completed the training also participated in the study. Reasons for not participating in the training were working nights or being on annual leave. Annual leave and night shifts were scheduled well in advance, before we asked house officers to participate. Nobody took the opportunity to discuss participation with a psychiatrist confidentially before the training, and nobody withdrew from the training after reading about the nature of the scenarios.

Demographics of the doctors who participated are reported in Table 1. The highest represented population was New Zealand Europeans in their first 2 years of clinical practice. At baseline, participants’ median WHO-5 score was 18 out of 25. The median score on the Rosenburg Self-esteem Scale was 29. Two participants had self-esteem in the low range, 11 in the medium range, and 10 in the high range.

**Table 1:** Participant demographics (*N* = 23)

Gender	
Female	10 (43%)
Male	13 (57%)
Other	0 (0%)
Ethnicity	
Asian	4 (17%)
Māori	2 (9%)
NZ European	16 (70%)
Pacific People	0 (0%)
Other	3 (13%)
Clinical Experience	
Postgraduate Year 1	10 (43%)
Postgraduate Year 2	12 (52%)
Postgraduate Year 3+	1 (4%)
Wellbeing and self-esteem	
World Health Organisation – Five Wellbeing Index (WHO-5)	Median 18 (IQR 15-20)
Rosenburg Scale	Median 29 (IQR 27-31)

### Quantitative Evaluation

After completing simulation training, doctors reported feeling more confident in managing a medical emergency in a psychiatric hospital (*p* < 0.001). Table 2 shows the median confidence reported by house officers before and after simulation training.

**Table 2:** Self-reported confidence before and after simulation-based learning

	Pre-simulation Median (% rated 4 or above)	Post-simulation Median (% rated 4 or above)	Statistical significance of change in median
How confident do you feel managing a medical emergency at a psychiatric hospital?	3 (4.2%)	4 (62.5%)	*p* < 0.001
How confident would you feel managing an attempted hanging at a psychiatric hospital?	2 (8.3%)	4 (50%)	*p* < 0.001
How confident would you feel managing post injection syndrome at a psychiatric hospital?	2 (0%)	4 (66.7%)	*p* < 0.001
How confident would you feel managing severe deliberate self-harm at a psychiatric hospital?	2 (4.2%)	4 (54.2%)	*p* < 0.001
How familiar are you with the emergency equipment available at the psychiatric hospital in which you work?	3 (8.3%)	4 (58.3%)	*p* < 0.001

They reported feeling significantly more confident managing post-injection syndrome, deliberate self-harm, and a non-fatal hanging (*p* < 0.001). They also reported being more familiar with the emergency equipment available at the psychiatric hospital where they worked.

### Qualitative Evaluation

Twenty-two out of 23 participants provided written feedback after completing the training. Participants wrote varied amounts of feedback in each text box provided. They wrote more in some text boxes while occasionally some text boxes were left empty. Table 3 outlines the codes and categories identified in the qualitative feedback. Participants reported it was helpful that the scenarios felt realistic. They reported that the scenarios were believable and something they might really have to manage during their rotations in psychiatry. They also reported that the immersive nature of the simulations was helpful. They reported that it was helpful to use the equipment which would be available in a real emergency. Many participants noted that having actors would improve the simulation fidelity. They also liked having real nurses involved in the simulations. “Experiencing the emergency” helped increase confidence to face similar real-life scenarios.

**Table 3:** Content analysis of participant feedback

Codes	Categories	Summary	Example Quotations
Realistic scenarios	Realistic Immersive Simulations	Simulations are most effective when the scenario is realistic, and the simulation is an immersive experience.	“The actual practise managing the emergency. Having nurses present, acting as nurses during the scenario. Having actors (Not just mannequins) [was most helpful]”
Immersive simulation			
			“Having to think on your feet. Communicating with other members of the simulation teaching. Figuring things out as you go.”
Psychiatry specific	Psychiatric Specific	Selecting psychiatric specific scenarios and exploring the psychological factors during the brief and debrief was unique and beneficial aspect of training.	“Range of scenarios covered, scenarios typically not seen at [general public hospital]”
Psychological debriefing			
			“All aspects, [were helpful] especially the practical aspects and trauma briefing.”
			“Debriefs gave us time to process and take learning out of the stress.”
Practising Danger, Response, Send for help, Airway, Breathing, Circulation (DRSABC)	Practising procedures	It was important to practise using the real equipment and be able to repeat these tasks until familiar.	“Practicing the motions helps”
Familiar with equipment			
Importance of repetition			
			“Proving to myself that the "system" (DRSABC) actually works; and that I can rely on it in an emergency.”
Safe environment	Safe environment experienced with peers.	Peer learning was more powerful than individual learning and occurred most effectively in a safe and contained training environment.	“Being able to observe how colleagues would handle specific scenarios and take pearls from them.”
Observing peers			
			“Helpful to have two [resident doctors] per scenario to bounce ideas off.”

Participants reported that having a psychiatric focus was helpful in two ways. Firstly, it was useful to experience scenarios that were more common in a psychiatric hospital compared to a general hospital. Secondly, they found the focus on psychological factors and trauma-informed care to be valuable and unique compared to previous training.

Learning was more powerful because it was done with peers. They reported that experiencing the simulations with their peers allowed participants to see how other doctors would manage the scenario. Some participants reported finding feedback from their peers particularly beneficial. They said this was facilitated by creating a safe and contained environment during the simulations and the debrief. During the debrief, participants were able to imagine changes in the scenario and talk through how this would impact their management. It was important to be “able to ask what if? and what next?”

Most participants reported that the program could be improved by having further simulation training during their psychiatric attachment. Repetition was seen as an important factor in making simulation training effective. “Repetition; it would be beneficial to continue completing these [simulations] at different times to reinforce knowledge and experience.”

## Discussion

This study informs the application of simulation-based learning for emotionally charged clinical scenarios and guides educators in developing confidence-building curricula. We demonstrate that simulation-based learning improves self-reported confidence to manage emergencies in a psychiatric setting. To our knowledge, this is the first study to simulate non-fatal hanging or non-suicidal self-injury on a psychiatric ward. These events can be both technically and psychologically challenging for clinicians. By replicating these highly emotive clinical events, this study allowed doctors to develop skills and confront challenging emotions in a safe and containing environment. Confidence to manage an attempted hanging is particularly important given its frequency and high lethality. Hanging is the most common method of suicide in mental health service users in New Zealand [20].

These findings build on previous studies that have evaluated simulation-based learning for the management of emergencies in a psychiatric setting. Thomson et al. [13] developed simulation training for new doctors managing behavioral disturbance and oversedation. Participants reported increased confidence after the training. Tong et al. [14] developed simulation-based learning for emergencies, including neuroleptic malignant syndrome, a critically physically unwell person with anorexia nervosa, and the management of a person with psychosis presenting with an indictable offense. Their simulations were completed by 19 junior doctors. The majority reported that the simulation had been helpful. Sharma et al. [15] provided Essential Life Support Training given by an intensive care physician to psychiatric trainees. They reported a significant increase in doctors’ confidence in managing emergencies following the simulation.

Simulation-based learning may be of particular benefit to doctors with less clinical experience in psychiatry [21]. A lack of emergency experience, the generalist nature of medical training, and the complexity of making rapid treatment decisions can contribute to less experienced doctors making errors [22]. Simulation-based learning has the potential to bridge the gap for new doctors entering clinical practice and ultimately improve the experience and outcomes of service users [21, 22].

It is important to acknowledge the emotionally strenuous nature of emergencies, and acute psychiatry more generally. Normalizing doctors’ vulnerability in these stressful situations is important in preserving psychological health and avoiding compassion fatigue [23, 24]. Simulation-based learning provides an opportunity to reflect and metabolize emotions when facing an emergency and has the potential to improve psychological resilience.

Social learning theory provides a framework for understanding why participants reported that learning was more impactful with peers [25]. In Bandura’s theory of social learning, attention is given to a peer’s behavior, where the observer anticipates doing it themselves and then reproduces the behavior with positive aspects being reinforced. Bandura theorized that when we observe others, we learn not only from their behavior but also from how other observers react to their behavior [25, 26]. In our study, house officers were able to learn by observing how their peers managed a simulated emergency and by observing the response of actors in the simulation. They could then anticipate their own behavior in a similar scenario before having an opportunity to practice in the subsequent simulation.

House officers who participated in this study appreciated the realistic and immersive nature of these simulations, and the containment provided in the brief and debrief. The importance of an immersive environment and effective debriefing in simulation-based learning is widely recognized [13]. Experiential learning theory is a helpful paradigm for understanding this observation [27]. In experiential learning theory, learners are exposed to a novel experience that involves risk. Learners actively experiment with the new concrete experience. Afterwards, critical reflection mediates meaningful learning [27]. While simulations are performed in a contained environment in our study, “experiencing the emergency” as novel and involving risk is important because it heightens the attention of house officers as they actively experiment with how to manage the emergency. To solidify learning, house officers were encouraged to critically reflect on the simulation during the debriefing.

A multidisciplinary approach to developing and implementing simulation training was a strength of this study. It allowed an authentic focus on interdisciplinary collaboration and communication. A natural expansion of this project would be to offer the training to other clinicians so that nurses and allied health professionals could also benefit from simulation-based learning. By exploring both the medical and psychological factors in each simulation, we were able to encourage a more nuanced understanding of complex clinical events. Facilitating the training in small groups allowed for greater openness in the debriefing. Psychiatric registrars were generally closer in age and experience to the house officers participating in the training. Their voice in the debriefing helped to bridge the gap between senior doctors and house officers. The social nature of our training and debriefing process potentially has advantages over virtual or AI driven simulations when considering emotional safety because facilitators and participants were able to recognize and adjust to each other’s social cues.

While psychological safety was not directly measured, it is reassuring for medical educators that no house officers opted out of this training due to fear of the psychological effects and no negative psychological effects were reported in written feedback. In fact, house officers wanted more simulation-based learning in this area.

A limitation of the study is that self-reported confidence may not reflect a house officer’s technical skills or proficiency to manage an emergency on a psychiatric ward; however, assessing performance in real-life scenarios was not a goal of this study. Future research would benefit from having co-designed scenarios by service users and clinicians. This could increase the authenticity of the simulations and enhance learning. In the future, we would consider having professional actors rather than clinicians in the role of those with mental illness and having oversight from those with lived experience to ensure the representation is fair and accurate. The qualitative component of the study provided a relatively superficial insight into the factors that impact learning because of the nature of data collection (by survey). An in-depth evaluation with semi-structured interviews or focus groups could increase the credibility of future research.

Future research would also benefit from service user involvement in designing scenarios. This collaboration would provide a deeper understanding of the psychological nuances involved and enhance the educational value of the training.

In conclusion, simulation-based learning allows training doctors to develop skills to manage complex scenarios or emergencies in a safe and containing environment. Our results demonstrate that simulation-based learning can improve house officers’ confidence managing emergencies in a psychiatric hospital setting. By exploring both the medical and psychological factors in each simulation, we were able to encourage a more nuanced understanding of complex clinical events. Further development of simulation-based learning within a psychiatric setting is justified.

## Data Availability

Data from this project will be made available upon reasonable request.
